# Epidemiological characteristics and spatiotemporal analysis of mumps at township level in Wuhan, China, 2005–2019

**DOI:** 10.1017/S0950268823000304

**Published:** 2023-02-27

**Authors:** Ying Peng, Peng Wang, De-guang Kong, Wen-zhen Li, Dong-ming Wang, Li Cai, Sha Lu, Bin Yu, Bang-hua Chen, Pu-Lin Liu

**Affiliations:** 1 Wuhan Centers for Disease Prevention and Control, Wuhan 430023, China; 2 School of Public Health, Tongji Medical College, Huazhong University of Science and Technology, Wuhan 430030, China

**Keywords:** Epidemiology, mumps, scan statistics, spatiotemporal clusters

## Abstract

The resurgence and outbreaks of mumps occur frequently in many countries worldwide in recent years, even in countries with high vaccination coverage. In this study, a descriptive and spatiotemporal clustering analysis at the township level was conducted to explore the dynamic spatiotemporal aggregation and epidemiological characteristics of mumps in Wuhan. During 2005 and 2019, there were 40 685 cases reported in Wuhan, with an average annual morbidity of 28.11 per 100 000 populations. The morbidity showed a fluctuating tendency, and peaked in 2010 and 2018. Bimodal seasonality was found, with a large peak between May and July, and a mild peak from November to January in the following year. Male students aged 5–9-year-old were the main risk group of mumps infection. Significant global spatial auto-correlation was detected except in 2007, 2009 and 2015. The spatial and temporal scan statistics indicated that the hot-spots mainly located at the western and southern areas of Wuhan with variations almost every year. Our findings could assist the public health authorities to develop and improve targeted health strategies, and allocate health resources rationally.

## Introduction

Mumps is an acute respiratory communicable disease caused by the paramyxovirus parotitis, which is highly contagious, especially among children [1]. The main clinical feature manifests as swelling of unilateral or bilateral parotid glands, accompanied with fever and pain. Although the common symptoms are mild in the majority cases, its complications, such as meningoencephalitis, pancreatitis, orchitis and oophoritis, may occur and can be serious [2].

The incidence of mumps reduced dramatically after the implementation of immunisation against mumps worldwide [3]. Nevertheless, the outbreaks and resurgence of mumps have been witnessed in many regions recently, even in those countries with high vaccination coverage of mumps-containing vaccine (MuCV), such as Portugal [4], United Kingdom [5], Canada [6] and United States [7]. According to the statistics of World Health Organization, the yearly reported number of mumps cases in the United States in 2016 and 2017 was almost twice the total number of mumps cases in the past 5 years. More than 119 000 mumps cases were reported in China in 2021, ranking the first among all countries and accounting for 53.27% of the total global cases [8]. These data indicated that mumps is still a serious public health problem globally and especially in China, and deserves further studies.

Describing the temporal–spatial distribution characteristics of diseases is one of the preliminary requirements for risk analysis. Spatial techniques, based on the geographic information systems (GIS), have been widely utilised in the field of health promotion and public health in the last few decades [9, 10]. Researchers usually applied spatial auto-correlation and space–time scan methods to probe the geographic distribution characteristics and to identify clusters for high- and low-risk regions and periods, contributing to the formulation and improvement of prevention and control measures [11–13].

There were a number of studies that have used the spatial analysis to explore the distribution of mumps in local regions of China, such as Chongqing city (western) [14], Guangxi province (southern) [15], Shandong province (northern coastal) [16] and Jiangsu province (eastern coastal) [17]. As far as we know, there was no study on the spatiotemporal characteristics of mumps in the central China. Wuhan, as the biggest central city in central China and with a relatively high incidence of mumps, the geography, social economy and climate of it differ from those of the areas mentioned above. Therefore, we carried out a spatiotemporal analysis at the township level in Wuhan between 2005 and 2019, to explore the epidemiological and spatiotemporal characteristics of mumps.

## Methods

### Study site

**Fig. 1.**
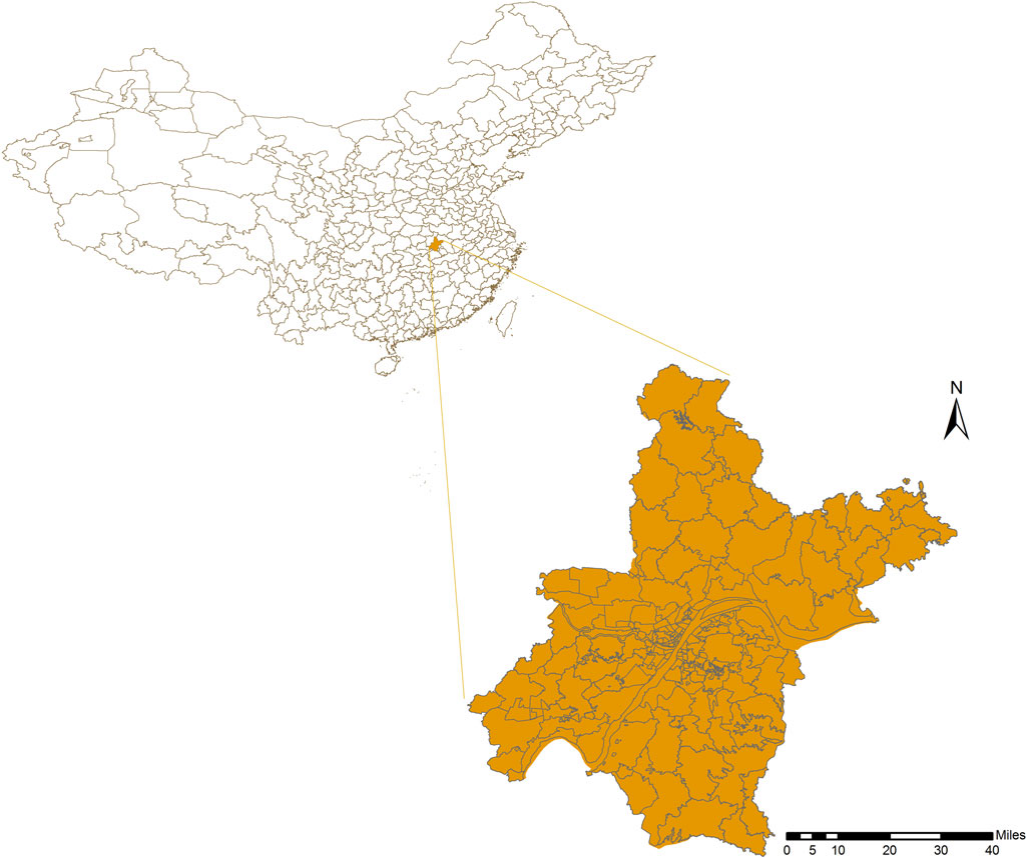
Geographic situation of Wuhan city in China.

Wuhan, the provincial capital city of Hubei province, is situated at 29°58′–31°22′N and 113°41′–115°05′E, where is the eastern part of Jianghan plain and the middle reaches of the Yangtze river (Fig. 1). It is an international wetland city, crisscrossing with rivers and hundreds of lakes. By the end of 2019, the city covers an area of approximately 8500 km^2^, with a permanent resident population of 12.33 million, and is known as an important science and education base, economic centre and comprehensive transportation hub in China.

### Data collection and management

The daily data on mumps cases that were reported from 2005 to 2019 in Wuhan city were retrieved from the National Monitoring and Reporting Management System for Communicable Diseases Information [18]. Mumps is classified as a class C notifiable disease, and all cases diagnosed by doctors in qualified hospitals at all levels throughout the country should be reported within 24 h through the system. The diagnosis of all mumps cases was made based on the clinical symptoms and laboratory test results according to the diagnostic guidelines for mumps issued by the Chinese Ministry of Health. The information of each reported mumps case includes case ID, gender, age, group classification, date of onset of the illness and residential address. The quality of data reporting is guaranteed by a series of measures, such as routine audits of data by the local Centers for Disease Control and Prevention (CDCs), regular training of doctors and field investigations of reporting accuracy and integrity. In this study, the obtained data were rechecked and the repeated cases were excluded.

For the purpose of performing spatial analysis, a town-level vector map of Wuhan was derived from the Chinese National Fundamental Geographic Information System. Demographic data were obtained from the Wuhan Bureau of Statistics. All mumps cases were geocoded by the specific administrative code and matched to the town-level layers of the polygons.

### Spatial auto-correlation analysis

This process included global and local spatial auto-correlation, and performed using GeoDa software (v1.12, Arizona State University, AZ, USA). The statistics of Moran's index (Moran's *I*) were utilised to investigate the global spatial auto-correlation. The values range from −1 to 1. A positive or negative value with statistical significance indicates positive or negative spatial auto-correlation, and a zero value means a random spatial auto-correlation. *Z*-scores and *P*-values were applied to determine the statistical significance of Moran's *I*. The local spatial auto-correlation can accurately detect specific aggregation patterns and regions, which is prompted by the local indicators of spatial auto-correlation (LISA). Four types of spatial auto-correlation can be seen in LISA maps: high–high (high-incidence areas surrounded by high-incidence regions); low–low (low-incidence areas surrounded by low-incidence regions); high–low (high-incidence areas surrounded by low-incidence regions) and low–high (low-incidence regions surrounded by high-incidence areas).

The spatiotemporal analysis data based on a township level were small area data. The results might be shaky barely relying on them. Spatial empirical Bayesian (SEB) was used to smooth the spatial variables to adjust for the fluctuation [19]. The algorithm of *k*-nearest neighbours was utilised to specify a spatial weights file to smooth the rates of each town. All auto-correlation analyses were on the basis of smoothing rate.

### Scan statistics

The Kulldorff's space–time scan statistics with a discrete Poisson model were performed in SaTScan™ software (v9.4) to detect the spatiotemporal clusters of mumps in Wuhan, 2005–2019. The basic concept of the scan statistics was to establish a scanning window which can move dynamically in space and time. The scanning window was defined by a cylindrical window, of which the bottom and height correspond to the circular (or elliptical) geographic base and the time length of potential clusters, respectively. For each scanning window, relative risk (RR) and log-likelihood ratio (LLR) were calculated to evaluate the increased risk within the window compared to outside. Significance was evaluated by 999 Monte Carlo simulations, and the statistically significant level was set as 0.05. The scanning window with the largest LLR was identified as primary cluster, and other scanning windows with statistically significant LLR were considered as secondary clusters.

The scanning statistical results would be very sensitive to the parameter settings [20]. For the setting of the maximum cluster size of space, we have tested the radius from 10 to 50%. From the results, we chose 15% as the optimal radius for the detected biggest cluster covering less than 15% of all the geographical areas [21]. The maximum cluster size of time was set as 50% of the overall study duration. All results of the scan analysis were visualised in ArcGIS software (v10.2, ESRI, Inc., Redlands, CA, USA).

## Results

### Demographic characteristics

**Fig. 2.**
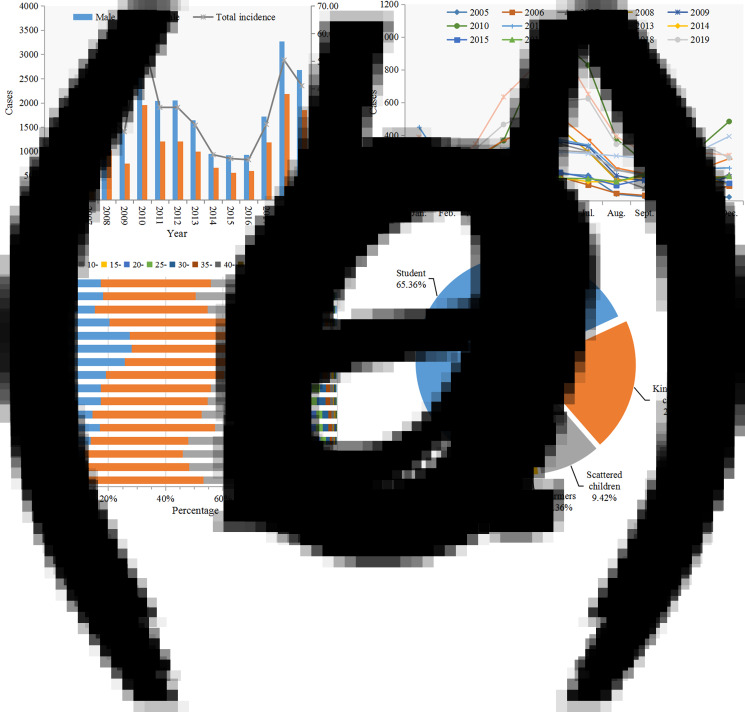
Epidemiological features of mumps in Wuhan between 2005 and 2019: (a) distribution of annual mumps incidence by gender; (b) monthly distribution of mumps; (c) mumps distribution by age and (d) mumps distribution by school age and occupation.

From 2005 to 2019, a total of 40 685 mumps cases were reported in Wuhan, with an average annual morbidity of 28.11 per 100 000. The epidemiological characteristics of mumps are shown in Figure 2. There were 25 163 males and 15 522 females cases, with the average sex-ratio 1.62. Cases of patients aged 5–9-year-old accounted for a majority (38.41% of all cases), followed by patients aged 10–14 (26.82%) and 0–4 (17.38%) years old (Fig. 2c). In terms of the school age and occupational distribution, students were the main group (65.36%), followed by preschool children (20.43%) (Fig. 2d).

### Temporal pattern

The changing tendency of mumps incidence in Wuhan during the study period consisted of three phases: the reported mumps incidence increased from 2005 to the peak in 2010 with a slight decrease in 2009; during 2011 and 2016, the trend was downwards; then it increased sharply again and reached to the second peak in 2018 (Fig. 2a). The seasonal trend was similar every year and two peaks were observed. The first large peak appeared between May and July, and the second small peak from November to January in the following year (Fig. 2b).

### Spatial distribution

**Fig. 3.**
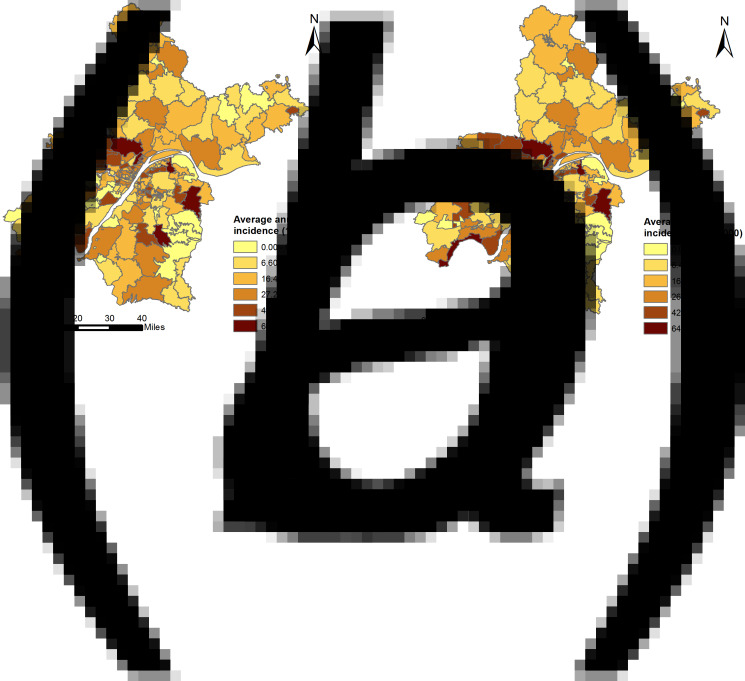
Average annual morbidity of mumps during 2005–2019 in Wuhan city: (a) the raw rate and (b) the smoothed rate.

**Fig. 4.**
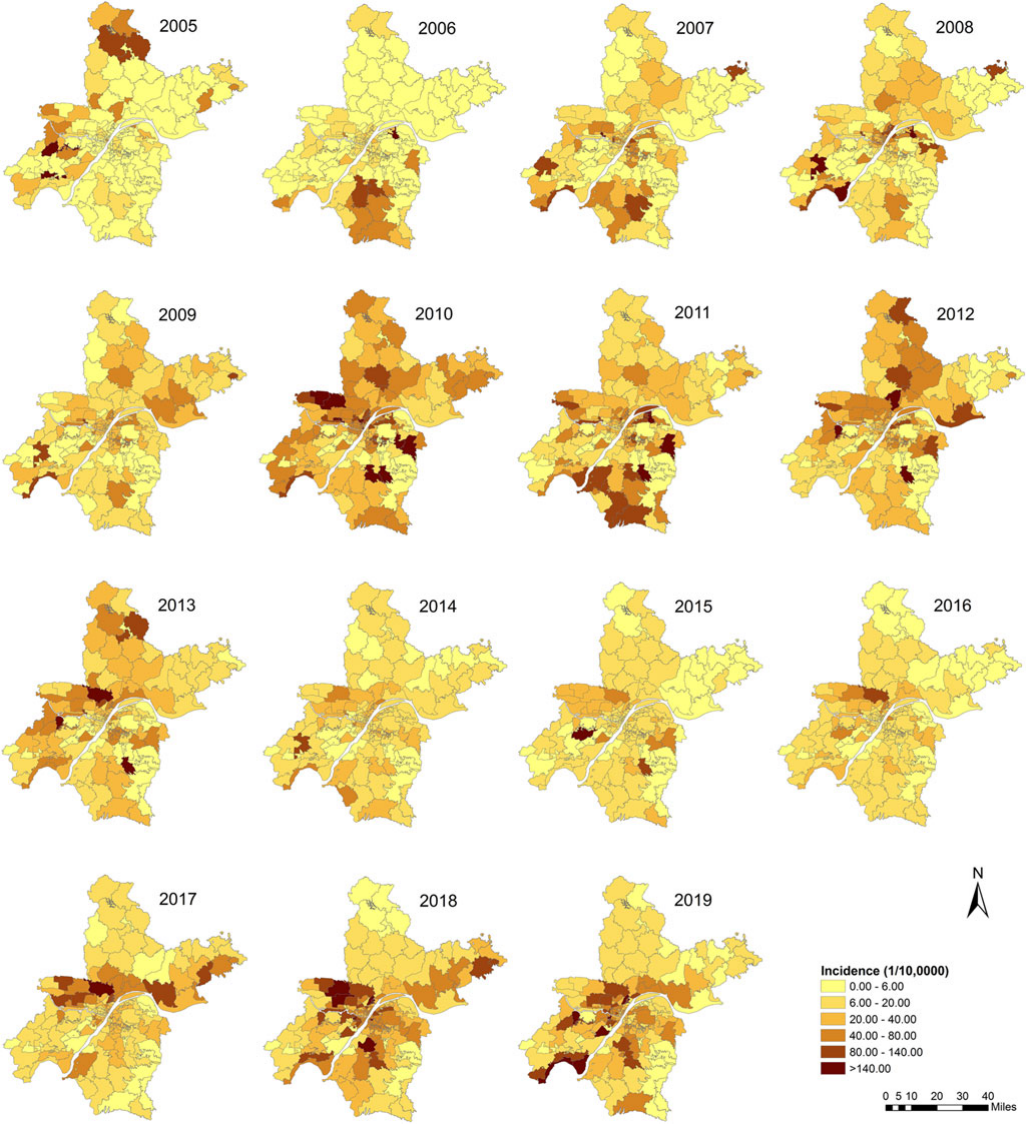
Annual morbidity of mumps at the town/street level in Wuhan city, China from 2005 to 2019.

The spatial distribution of annual incidence rate of mumps is shown in Figure 3. It indicated that the prevalence of mumps was heterogeneously distributed at the township level. The central belt and south part had high incidence, while the north part had relatively low incidence. Significant difference of the smoothing rate with SEB compared to the raw rate was not observed. Both of them showed almost the same pattern. The thematic map showed a similar geographical distribution pattern that the high incidence regions in Wuhan city changed over years but mainly concentrated in the central and western areas (Fig. 4).

**Fig. 5.**
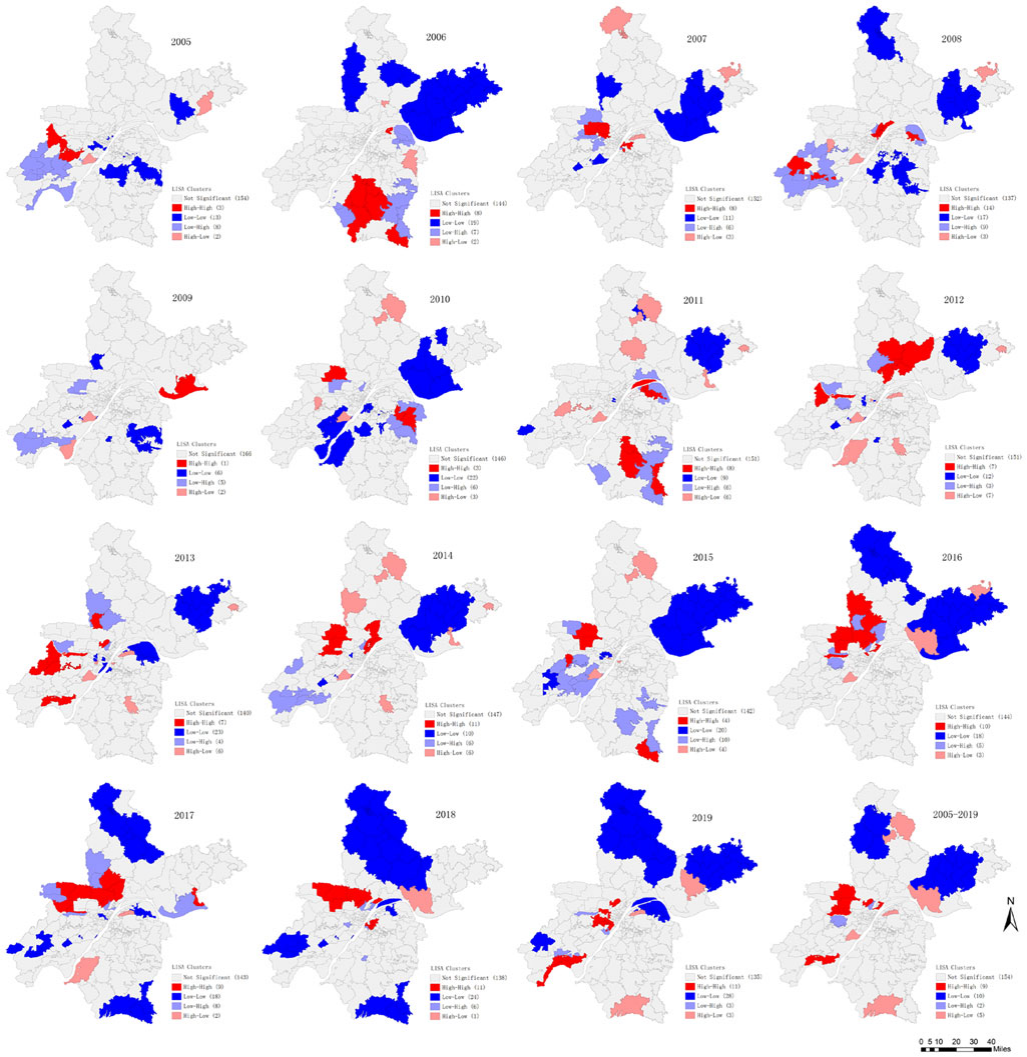
Yearly local spatial auto-correlation of mumps incidences at the town/street level in Wuhan city, China from 2005 to 2019.

The global spatial auto-correlation test showed a significant positive spatial auto-correlation for the average annual mumps morbidity in Wuhan (Table 1). Similar statistically significant results were also detected when the analyses were performed yearly, except in 2007, 2009 and 2015. Nine hot-spots and ten cold-sports of the average annual incidence were identified by the LISA analysis. The yearly cluster pattern of LISA is presented in Figure 5, and the hot-spots were mainly concentrated in the western and southern parts of Wuhan with slight transfer yearly. As listed in Table 2, 2008 was the year with the most hot-spots of 14 towns. Cold-spots were mainly distributed in the northern part of Wuhan.

**Table 1.** Results of global spatial auto-correlation test of mumps in Wuhan, China, 2005–2019

Year	Moran's *I*	*E*[*I*]	Mean	s.d.	*Z*-score	*P*-value
2005	0.0728	−0.0059	−0.0046	0.0351	2.2046	0.027
2006	0.0385	−0.0059	−0.0068	0.0238	1.9006	0.046
2007	0.0279	−0.0059	−0.0058	0.033	1.0216	0.144
2008	0.1682	−0.0059	−0.0046	0.0379	4.5645	0.001
2009	−0.0214	−0.0059	−0.0054	0.0399	−0.4009	0.365
2010	0.1295	−0.0059	−0.004	0.0406	3.287	0.004
2011	0.1032	−0.0059	−0.0065	0.0393	2.7964	0.013
2012	0.0826	−0.0059	−0.0056	0.0401	2.2011	0.019
2013	0.0954	−0.0059	−0.0061	0.0394	2.5748	0.011
2014	0.0648	−0.0059	−0.0073	0.0394	1.8308	0.042
2015	0.0147	−0.0059	−0.0086	0.0349	0.6649	0.221
2016	0.1348	−0.0059	−0.0064	0.0384	3.6783	0.002
2017	0.1625	−0.0059	−0.0066	0.0375	4.5111	0.001
2018	0.1897	−0.0059	−0.006	0.0414	4.7211	0.001
2019	0.1612	−0.0059	−0.0063	0.0401	4.1796	0.002
Average	0.0709	−0.0059	−0.0055	0.0408	1.8723	0.037

*E*[*I*], the theoretical mean of Moran's *I* statistic; mean and s.d., the centralised and discrete trends of simulated empirical distribution, respectively.

**Table 2.** High–high areas (hot-spots) detected in the LISA analysis for mumps from 2005 to 2019

Year	towns/streets (*n*)	Hot-spot lists
2005	3	Zhangwan, Caidian, Daji
2006	8	Qingshanzhen, Gongrencun, Husi, Anshan, Wulongquan, Zhengdian, Zhifang, Shidong
2007	8	Jinghe, Wujiashan, Jinyinhu, Changfeng, Shouyi, Zhonghualu, Jiyuqiao, Shuiguohu
2008	14	Zhurushan, Tonghubanshichu, Tangjiadun, Huaqiao, Houhu, Laodong, Chezhan, Yongqing, Erqi, Xincun, Houhu, Baibuting, Danshuichi, Shenjiaji
2009	1	Shuangliu
2010	3	Baoxie, Jiufeng, Boquan
2011	8	Shuan, Wulongquan, Zhifang, Baiyushan, Changqian, Gongrencun, Qingshanzhen, Tianxing
2012	7	Zhangwan, Cihui, Shekou, Sanliqiao, Datanbanshichu, Qianchuan, Liuzhi
2013	7	Dongjing, Suohe, Yuxian, Zhangwan, Cihui, Jiangjunlu, Tianhe
2014	11	Cihui, Changqing, Jinghe, Boquan, Laodong, Erqi, Xincun, Houhu, Danshuichi, Shenjiaji, Shekou
2015	4	Husi, Caidian, Jinghe, Boquan
2016	10	Cihui, Tangjiadun, Xincun, Danshuichi, Jiangjunlu, Jinghe, Boquan, Panlongcheng, Hengdian, Qijiawan
2017	9	Changqing, Zoumaling, Boquan, Dongshan, Jiangjunlu, Panlongcheng, Shekou, Hengdian, Zhangduhu
2018	11	Huanghelou, Zhonghualu, Shouyilu, Zhongnanlu, Shuiguohu, Shenjiaji, Jiangjunlu, Jinghe, Dongshan, Boquan, Panlongcheng
2019	11	Dengnan, Dongjing, Jiangdi, Longyang, Wulidun, Qinduankou, Jianghanerqiao, Yongfeng, Zongguan, Changfeng, Jiangjunlu
Average	9	Dongjing, Xincun, Danshuichi, Jiangjunlu, Cihui, Changqing, Wujiashan, Jinghe, Boquan

### Spatiotemopral clustering analysis

**Fig. 6.**
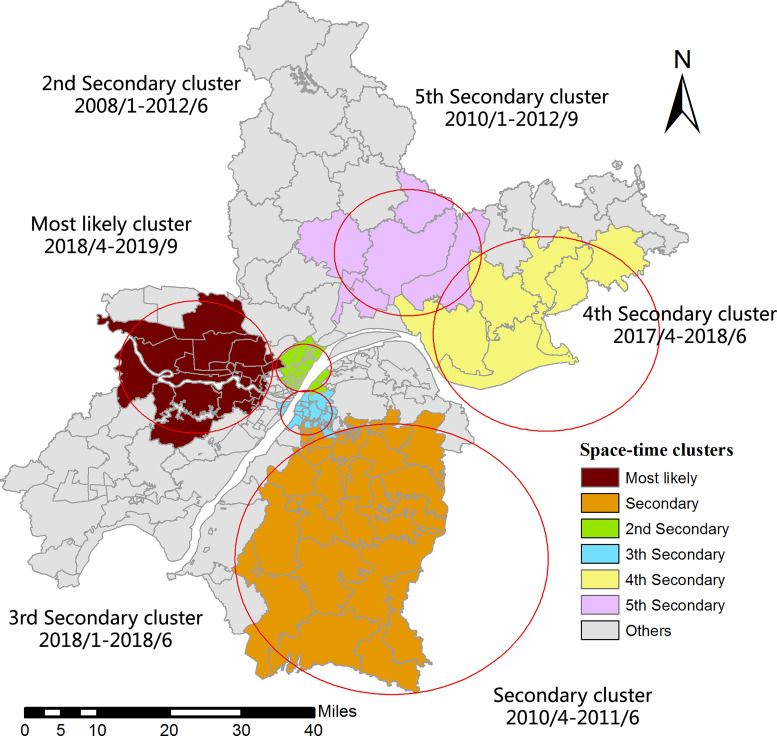
Spatiotemporal clusters of mumps incidences at the town/street level in Wuhan city, China between 2005 and 2019.

The results of spatiotemporal scan statistics showed that mumps in Wuhan was non-randomised distributed in time and space, and six high-incidence clusters of mumps were identified. The most likely cluster, consisting of 23 adjacent towns/streets along the Han-Jiang river, was located in the west and central areas of Wuhan from April 2018 to September 2019 (RR = 3.11, LLR = 875.00, *P* < 0.001). The detailed results are mapped in Figure 6 and listed in Table 3.

**Table 3.** Clusters identified by the space–time scan statistics on mumps incidences in Wuhan, China, 2005–2019

Cluster pattern	Range of cluster time	Coordinates/radius	Towns/ streets (*n*)	Observed cases	Expected cases	RR	LLR	*P*-value
Most likely	2018/4/1 to 2019/9/30	(30.63N, 114.09E)/14.73 km	23	1963	651.65	3.11	875.00	<0.001
Secondary	2010/4/1 to 2011/6/30	(30.24N, 114.48E)/30.15 km	23	1552	472.47	3.38	780.92	<0.001
2nd secondary	2008/1/1 to 2012/6/30	(30.62N, 114.30E)/5.29 km	23	3093	1502.44	2.15	675.47	<0.001
3rd secondary	2018/1/1 to 2018/6/30	(30.53N, 114.31E)/4.94 km	15	694	193.55	3.63	388.86	<0.001
4th secondary	2017/4/1 to 2018/6/30	(30.69N, 114.79E)/21.68 km	7	614	191.88	3.23	294.24	<0.001
5th secondary	2010/1/1 to 2012/9/30	(30.86N, 114.51E)/14.08 km	6	690	284.68	2.45	207.59	<0.001

## Discussion

The MuCV was administrated on a voluntary and self-paid basis in Wuhan before 2008. Children aged 5–10 years might have not received the MuCV for the short time of MuCV introduction, and therefore became susceptible to mumps infection. Thus, despite one dose of MuCV being integrated into the expanded national immunisation programme and routinely offered free of charge to children aged 18–24 months in 2008, the incidence of mumps in Wuhan was increasing continuously from 2005 to 2010, which was the same as the tendency of mumps incidence in the whole country [22]. The trend of mumps incidence from 2011 to 2016 was downwards, indicating that the control and preventive measures were effective. Several studies have shown that the concentrations of mumps-specific antibody decreased and the immunity waned over time [23, 24]. An upward trend of mumps incidence occurred again in 2017, which would be attributed to the accumulation of susceptible populations due to attenuated vaccine effectiveness.

Studies on mumps in other cities have found that the incidence in males was significantly higher than that in females [14, 25], which was the same as our findings. These findings could be explicated as different patterns of behaviour between males and females [26]. Males were more active relatively and paid less attention to hygiene than girls, which would increase their exposure to the mumps virus and facilitate mumps transmission. The present study found that students and kindergarten children, and especially children aged 5–9-year-old were the main groups. A prospective study in children aged 3–7 years demonstrated that seropositivity of antibodies against mumps was decreased over time and would be only 58.18% after 3 years of vaccination [27]. Besides, the seroprevalence of children received one-dose MuCV was much lower than that of children received two-doses of MuCV [28]. Indeed, the morbidity of mumps in cities such as Beijing and Shanghai where two-doses of MuCV have been administered was much lower than that in other cities [22]. Hence, more than one dose of MuCV should be introduced to the immunisation schedule as children vaccinated single dose of MuCV at 18–24 months of age were still at high risk of mumps infection during kindergarten and primary school.

Mumps onset in Wuhan displayed a bimodal seasonal distribution, with a large peak between May and July, and the second small peak from November to January in the following year. Double seasonal peaks were also found in some areas of China [17] and also observed in other communicable diseases such as hand-foot-mouth diseases [29]. The peak periods coincided with the school semesters. The crowded and confined environment of schools and kindergartens facilitates the spread of mumps. Therefore, kindergartens and schools should strength the management of students’ health and education, and it is recommended to check the immunisation certificates at admission, provide emergent inoculation to non-vaccinated students.

The spatial auto-correlation analyses results combining with the thematic map in this study implied that the spatial distribution of mumps in Wuhan was significantly heterogeneous at the township level in 2005–2019. Socio-economic levels, climate and health care services might be potential elements affecting this regional variation. Zhang's research team found that the meteorological factors such as temperature, humidity and wind velocity exerted significant impact on the incidence and transmission of mumps [30]. Another study in mainland China reported that the trend of mumps incidence varied with different economic levels [31]. Although no similar study has been conducted to analyse the exact association between mumps prevalence and climatic and social economic factors in Wuhan, the potential correlation could be interpreted by the spatiotemporal distribution of mumps in our study. The most likely cluster found by the elliptical window scanning was located in the western part of Wuhan, where is distant suburban area and along the Han-jiang river. The secondary cluster was found in the southeastern of Wuhan, where is also distant suburban area and around the Liang-zi lake, the second biggest lake of Hubei. Hot-spots identified by LISA analyses were in accordance with the results of scan statistics. The climate of these regions adjacent to rivers or lakes during the mumps epidemic season is relatively warm and humid, which is advantageous for virus transmission [32]. Besides, it is widely known that the economy of distant suburban regions is less developed than that of the central areas in Wuhan. Therefore, it is recommended to improve the health care services by providing health interventions such as carrying out health education, allocating health resources rationally and increasing the rate of MuCV immunisation among risk population in these high-risk areas.

The findings of our study could help public health policy-makers to formulate and improve control and prevention policies and strategies of mumps, and give some clues for profound researches on the influencing factors for mumps epidemics. Meanwhile, there are some limitations of this study. First, the data were obtained from a passive monitoring system. The mumps cases might be underestimated because of unreported mild cases with sub-clinical symptoms. Second, this study only analysed the epidemiological and spatiotemporal characteristics of mumps. The actual potential socioeconomic and environmental factors which may be contributed to the mumps epidemics still need further investigation and explanation.

## Conclusions

To the best of our knowledge, this study first described the epidemiological characteristics of mumps with usage of spatial techniques based on GIS, and detected the high-risk clusters and hot-spots in space and time at the township level in Wuhan, China between 2005 and 2019. The prevalence showed a fluctuating pattern with the periodicity of 5 years and a bimodal seasonality. Male students aged 5–9-year-old were high-risk groups of mumps. A two or three doses of MuCV vaccination should be incorporated into the routing immunisation procedure to obtain higher and more durable immunity. The high-risk regions mainly distributed in the western and southern part of Wuhan. Considering the areas of hot-spots varied almost yearly, it is necessary to conduct real-time spatial surveillance and early warning to trace the high-risk regions.

**Table:** 

**Panel 1: Some concepts and formula for calculation** Annual incidence per 100 000 = (annual number of cases/annual number of population) × 100 000 Average annual incidence per 100 000 = (the total number of cases/the total number of population) × 100 000 Average sex ratio = the total cases of male/the total cases of female

## Data Availability

The data are available from the authors with reasonable request.
